# Supplementary material for: Healthcare Providers’ Knowledge and Attitude Towards Abortions in Thailand: A Pre-Post Evaluation of Trainings on Safe Abortion
Source: Int J Environ Res Public Health. 2020 May 4;17(9):3198. doi: 10.3390/ijerph17093198 (PMC7246465; doi:10.3390/ijerph17093198)
Supplement: Supplementary file 1 [file ijerph-17-03198-s001.pdf]

Supplementary Material

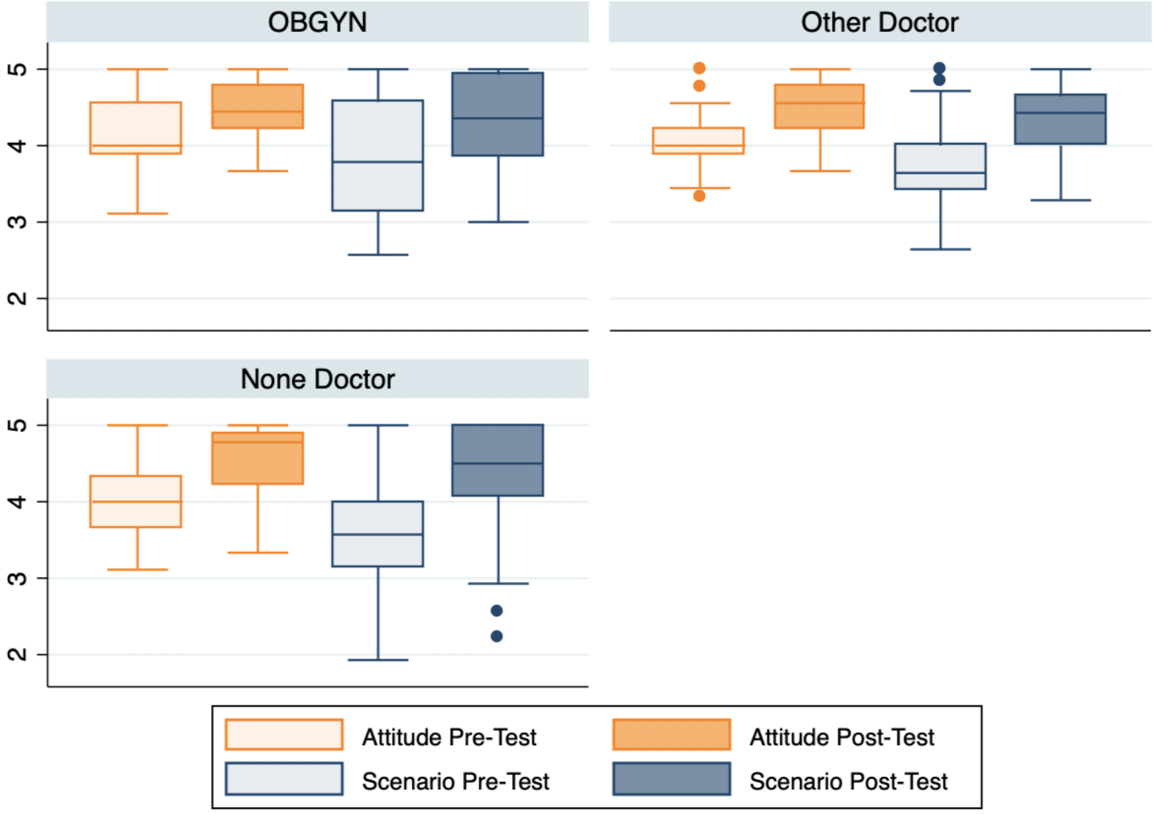

.

Figure S1. Box Plot of the Average Pre-test and Post-test Response by career type.

Table S1. Regularized least squares analysis on towards adolescents and women unplanned for pregnancy, and unsafe abortions.

(Reference: OBGYN)	Coef. (95% CI)	*p*-Value
Non-OBGYN doctor	0.0183 (−.042, .125)	0.710
Non-Doctor	0.0953 (0.056, 0.147)	0.070
Prior knowledge of regulation	−0.060 (−0.074,−0.050)	0.291
Experience in treating	−0.064 (−0.122,−0.005)	0.335
Experience in counselling	0.000 (0.000, 0.001)	0.617

Table S2. Regularized least squares analysis on abortion scenarios.

(Reference: OBGYN)	Coef. (95% CI)	*p*-Value
**Non-OBGYN doctor**	−0.056 (−0.078,−0.013)	0.314
**Non-Doctor**	0.162 (0.122, 0.221)	0.006
**Prior knowledge of regulation**	0.005 (−0.033, 0.037)	0.938
**Experience in counselling**	0.000 (0.000, 0.001)	0.428
